# Environmental impact of the COVID-19 pandemic – a lesson for the future

**DOI:** 10.1080/20008686.2020.1768023

**Published:** 2020-05-25

**Authors:** Mohamed E. El Zowalaty, Sean G. Young, Josef D. Järhult

**Affiliations:** a Zoonosis Science Center, Department Of Medical Biochemistry and Microbiology, Uppsala University, Uppsala, Sweden; b Department of Environmental and Occupational Health, University of Arkansas for Medical Sciences, Little Rock, AR, USA; c Zoonosis Science Center, Department of Medical Sciences, Uppsala University, Uppsala, Sweden

**Keywords:** COVID-19, pandemic, SARS-CoV-2, coronaviruses, environment, impact, infectious diseases, health

## Abstract

The environment is an integral component of human and animal health. COVID-19 is a global health challenge in the twenty-first century. The emergence of SARS-CoV-2 in Wuhan, China in December 2019, and its spread to regional countries and nowadays affecting more than 210 countries worldwide represents the first pandemic in history to be caused by a coronavirus. The COVID-19 pandemic has huge impacts on most aspects of human activities, as well as on the economy and health care systems. Lock-downs, quarantines and border closures in the wake of the pandemic have led to reductions in air pollution through decreased travel and production. These positive environmental effects are likely mostly temporary, but may serve as an example that changes in our way of life can have prompt positive effects for the environment and demonstrate the usefulness of travel-reducing measures such as teleconferencing. Thus, acknowledging that COVID-19 is first and foremost a global disaster, the pandemic may inspire to future behavioral changes with positive environmental effects.

To date, COVID-19 continues to be a challenge to global public health. The newly emerging SARS-related coronavirus designated as SARS-CoV-2 is the third highly pathogenic *Betacoronavirus* to infect human populations in the twenty-first century [1]. SARS-CoV-2 emerged in Wuhan, China in late December 2019 and has spread to more than 210 countries in every continent on earth except Antarctica. COVID-19 is the first pandemic in history to be caused by a coronavirus. While the huge negative effects of living through the COVID-19 pandemic are obvious – psychological stress, fear, severe global economic losses, overwhelmed health care systems and general disruption of societies – the ongoing pandemic may also have some indirect positive impacts. Virus outbreaks often have large negative impacts on human health [2] but changes in our way of life due to outbreak responses may serve as demonstrations of possible positive changes for the environment, health of humans, animals and the ecosystem.

Social distancing directives and quarantines have led to substantial decreases in commuting travel as many jobs shift to working from home. The widespread quarantines and travel restrictions imposed by several countries have resulted in reduced use of and demand for oil and its products, which has resulted in reduced emissions of smoke and waste due to oil consumption. For example, the National Aeronautics and Space Administration (NASA) and the European Space Agency (ESA) recently reported that nitrogen dioxide air pollution has been significantly reduced in connection with the community quarantine and lockdown in Wuhan and other cities in China [3]. The Centre for Research on Energy and Clean Air also reported that CO2 emissions in China were down 25% in the two weeks following the Chinese New Year holiday [4].

Air pollution affects climate and may induce drastic changes on ecosystems, which can also exacerbate infectious diseases outbreaks by affecting pathogens, hosts, vectors, and transmission dynamics [5,6]. Air quality in China has been reduced in the past few years and this has resulted in increased hospitalizations due to respiratory diseases [7]. The country’s response to COVID-19 may have at least partially slowed this trend.

International air travel has also decreased dramatically since the onset of the COVID-19 outbreak due to the implementation of travel restrictions. Recently, extended travel restrictions have been implemented all over the world. In March 2020 and in the midst of the COVID-19 global public health emergency, more regional quarantine and lockdown measures are also being rolled out in several areas such as the US, EU, the Middle East and North Africa region, Asia, and Australia. Most likely, these actions will have similar positive effects on reducing air pollution, at least temporarily, as observed in China through reduced use of fossil fuels and reduced discharge from production systems.

The COVID-19 pandemic is first and foremost a global health emergency with severe consequences for health and economy, but it can also serve as an example that changes in travel and production quickly and distinctly can improve air quality, and can reduce the carbon footprint which will translate to improved environmental health. It remains to be seen to what extent the changes brought on by the pandemic, such as increases in telecommuting and reduced travel, will remain once the immediate threat has passed.

Obviously, the dramatic actions taken during the pandemic cannot be directly copied in non-pandemic times to achieve the positive benefits. However, we believe that lessons can be learned, and that inspiration can be gained from the fact that quick positive feedback is seen when action is taken.
